# Antipsychotic Prescribing in an Older Persons Crisis Team. Has Adherence to the Guidelines Improved Since the Implementation of a Care Pathway for Managing Behaviour That Challenges in Dementia?

**DOI:** 10.1192/bjo.2025.10661

**Published:** 2025-06-20

**Authors:** Roxy Celyn Rees, Arpita Chakrabarti

**Affiliations:** 1 Cardiff University, Cardiff, United Kingdom; 2 University Hospital Llandough, Cardiff, United Kingdom

## Abstract

**Aims:** Behavioural and psychological symptoms (BPSD) such as agitation and psychosis, are a common challenge faced in the management of dementia. Despite NICE guidelines prioritising non-pharmacological interventions, according to an audit conducted in 2022, antipsychotics were frequently used first-line by the React team at University Hospital Llandough, raising safety concerns. Following this audit, a care pathway for managing challenging behaviour in dementia was implemented. This study aims to evaluate adherence to BPSD management guidelines and assess improvements compared with the 2022 audit.

**Methods:** This is a retrospective audit that includes all patients referred to the React team between June 2023 and May 2024 with a dementia diagnosis and prescribed antipsychotics for BPSD. Data was extracted from case notes using the PARIS database, guided by Oxford Health’s BPSD management recommendations, derived from NICE Guideline 97. Information gathered includes dementia type, consideration of other causes for presenting symptoms, use of non-pharmacological methods, antipsychotic prescribing practices, and adherence to monitoring guidance. Results were compared with the 2022 audit using chi-square tests to assess statistically significant differences.

**Results:** 40 patients (mean age: 81, range 68–95) were included and compared with 73 (mean age: 79, range 63–95) from the 2022 audit. Alzheimer’s disease accounted for 30% of cases, while 33% had unspecified dementia. Consideration of other causative factors was documented in 23% of cases, with treatment provided in 20%. Non-pharmacological approaches were utilized in 35% of cases, a substantial increase from 1% in 2022 (χ2 (1,113) = 25.386, p<0.001). Antipsychotics were used first-line in 65% of cases compared with 99% in 2022. Risperidone was prescribed in 75% of cases, and 85% were started on the lowest dose (χ2 (1,102) = 10.891, p<0.001). Monitoring adherence improved from 12% to 45% (χ2(1,113) = 15.168, p<0.001).

**Conclusion:** Since the implementation of the care pathway there has been increase in non-pharmacological interventions, appropriate dosing, and monitoring of antipsychotic use. However, there was no significant improvement in considering and treating other potential causes for symptoms, and documentation gaps persist. To enhance guideline adherence, React’s processes for assessing underlying causes and documenting patient management require review. A checklist in patient notes could further standardise care and ensure comprehensive documentation. Collaboration with primary care and memory services is essential to prioritise early-stage non-pharmacological interventions, potentially reducing crises and antipsychotic reliance. Further studies are needed to evaluate long-term outcomes of these initiatives.

